# A Video-based Debriefing Program to Support Emergency Medicine Clinician Well-being During the COVID-19 Pandemic

**DOI:** 10.5811/westjem.2020.8.48579

**Published:** 2020-09-25

**Authors:** Derek L. Monette, Wendy L. Macias-Konstantopoulos, David F.M. Brown, Ali S. Raja, James K. Takayesu

**Affiliations:** Massachusetts General Hospital and Harvard Medical School, Department of Emergency Medicine, Boston, Massachusetts

## Abstract

**Introduction:**

Emergency clinicians on the frontline of the coronavirus pandemic experience a range of emotions including anxiety, fear, and grief. Debriefing can help clinicians process these emotions, but the coronavirus pandemic makes it difficult to create a physically and psychologically safe space in the emergency department (ED) to perform this intervention. In response, we piloted a video-based debriefing program to support emergency clinician well-being. We report the details of our program and results of our evaluation of its acceptability and perceived value to emergency clinicians during the pandemic.

**Methods:**

ED attending physicians, resident physicians, and non-physician practitioners (NPP) at our quaternary-care academic medical center were invited to participate in role-based, weekly one-hour facilitated debriefings using Zoom. ED attendings with experience in debriefing led each session and used an explorative approach that focused on empathy and normalizing reactions. At the end of the pilot, we distributed to participants an anonymous 10-point survey that included multiple-answer questions and visual analogue scales.

**Results:**

We completed 18 debriefings with 68 unique participants (29 attending physicians, 6 resident physicians, and 33 NPPs. A total of 76% of participants responded to our survey and 77% of respondents participated in at least two debriefings. Emergency clinicians reported that the most common reasons to participate in the debriefings were “to enhance my sense of community and connection” (81%) followed by “to support colleagues” (75%). Debriefing with members of the same role group (92%) and the Zoom platform (81%) were considered to be helpful aspects of the debriefing structure. Although emergency clinicians found these sessions to be useful (78.8 +/− 17.6) interquartile range: 73–89), NPPs were less comfortable speaking up (58.5 +/− 23.6) than attending physicians (77.8 +/− 25.0) (p = < 0.008).

**Conclusion:**

Emergency clinicians participating in a video-based debriefing program during the coronavirus pandemic found it to be an acceptable and useful approach to support emotional well-being. Our program provided participants with a platform to support each other and maintain a sense of community and connection. Other EDs should consider implementing a debriefing program to safeguard the emotional well-being of their emergency clinician workforce.

## INTRODUCTION

The 2019 coronavirus pandemic poses unique systems and psychological challenges to clinicians in the emergency department (ED). Clinicians involved in managing public health crises are prone to experiencing a range of emotions including anxiety, fear, and grief that can lead to disaster-related distress.^1^–^3^ This has become increasingly evident as the pandemic continues, and these reactions impact the resilience and retention of the ED workforce.^4^,^5^

Critical incident stress debriefing (CISD) is a recommended practice for processing clinician reactions and may reduce the incidence of disaster-related distress.^6^,^7^ It is likely most effective when performed as soon as possible in time and place to an event.^7^,^8^ However, the coronavirus pandemic demands that emergency clinicians balance a variety of stressors while on shift including high acuity, patient surge, and risks to personal physical safety. In response, we designed and implemented a video-based ED debriefing program to support the well-being of our emergency clinicians. Our program had the following objectives: 1) to facilitate discussion regarding emotional reactions to coronavirus disease 2019 (COVID-19); 2) to provide peer-to-peer support in an era of social distancing; and (3) to identify resources to improve self-care and build resilience. The objective of this paper was to describe the details of our program and report the results of our evaluation of its acceptability and initial impact on emergency clinicians providing care during the coronavirus pandemic.

## METHODS

### Design and Participants

Our program was offered to emergency clinicians at our quaternary-care academic medical center that sees over 110,000 ED annual visits. The staff includes 119 physicians (attendings and residents) and 50 non-physician practitioners (NPP) (physician assistants and nurse practitioners). An invitation was sent to ED attending physicians, resident physicians, and NPPs by email to participate in voluntary debriefings on well-being and emotional reactions to COVID-19. To increase psychological safety, the email stated that each session would be for a single clinician role group (eg, attending physicians only) and identified the facilitators (DLM and JKT, both present for all sessions).^9^ The email provided a link to the secure, password-protected Zoom meeting. Our hospital’s institutional review board (IRB) approved evaluation of this program.

### Facilitator Experience

The same two ED faculty (DLM and JKT) with experience in clinical debriefing, simulation, and clinician wellness co-facilitated each session. In the year preceding this debriefing program (2018–2019), these two facilitators completed >150 hours of debriefings with ED staff in individual or team-based medical simulations. Both facilitators have received formal training in group debriefing at the Center for Medical Simulation (Boston, MA) and through Master of Science coursework. Finally, JKT has 15 years of experience in residency leadership (2003–2018), during which time he focused on resident wellness, mentorship, and professional development. These experiences informed study design and debriefing structure.

### Debriefing Structure

Two ED attendings with experience in clinical debriefing, simulation, and clinician wellness co-facilitated each session. We selected a co-facilitator approach so that facilitators could support each other in their own emotional reactions to the debrief and model normalizing statements for participants. We also employed a “follow the leader” co-debriefing strategy.^10^ An advantage of this strategy is that one attending can primarily lead the debriefing while the other observes participants for reactions, non-verbal cues, and communicates with the lead via Zoom’s chat function.^10^ The facilitators huddled before each session to identify any particular topics that the group might benefit from debriefing (eg, a recent surge in patient volume).

Participants were asked to log in from a non-clinical environment, use video and headphones, and attest to confidentiality of participation at the start of each session, which were divided into three phases (Appendix A):

1. Opening (5 minutes): The facilitators outline the objectives, describe a confidentiality contract, and discuss a plan for maintaining a psychologically safe environment. We informed participants that we would not record the audio or video of these sessions, and would not provide a list of participants to departmental leadership. We reiterated that solving clinical systems or operational problems is outside the scope of the debriefing, but with participant permission, we would submit concerns that came up during the debriefing to departmental leadership in a de-identified manner. Finally, we informed participants that Zoom has a “lobby” function, or private virtual space, in which one can take a break from the call if distressed without leaving the session altogether.
2. Discussion (45 minutes): The facilitators prompt reflection on emotional reactions to recent events in the ED or at home, steering the discussion toward empathic validation, normalizing reactions instead of problem solving. Facilitators often modeled these statements at the start of this phase as an “ice-breaker,” and communicated with participants using the chat function in addition to the video.
3. Closing (5 minutes): The participants have an opportunity to share any final burning issues; the group develops 1–2 major take-aways from the session; and facilitators share a link to a list of well-being resources provided by the hospital.

After each session, facilitators debriefed each other on their own reactions to the session and summarized any specific systems-based or operational concerns approved by the participants to be shared with departmental leadership.

### Survey Design and Analysis

An anonymous and voluntary 10-point survey was distributed electronically to all participants at the end of the pilot (Appendix B). To create this survey, study team members (DLM, JKT) reviewed previous evaluations of debriefing and peer-support programs related to well-being in healthcare, including survey-based studies.^11^–^13^ Based on these results, study team members (DLM, JKT) created questions that focused on debriefing participants’ experience with the program. For multiple-answer questions (3, 5, and 8), we pre-defined a significant result to be a choice that >70% of respondents included in their answer. We selected these answer choices based on the results of previous evaluations of debriefing programs and our program objectives.^11^–^13^ Questions 4, 6, and 7 asked participants to rate the relative utility of these sessions and comfort speaking up during a debriefing using a visual analogue score (VAS).^14^ Finally, we solicited feedback from remaining study authors and incorporated recommendations into the final survey.

The mean and interquartile range (IQR) were determined for each role group. Remaining questions were single option or open-ended. We used SurveyMonkey Inc. (San Mateo, California) to compile survey data and performed our analysis using Microsoft Excel (Microsoft Corporation, Redmond, WA).

## RESULTS

We completed 18 debriefing sessions between March–April 2020 with 68 emergency clinicians (29 attending physicians, 6 resident physicians, and 33 NPPs). The mean number of participants in each session was 8.5 (IQR 6–10) for attendings, 4 (IQR 3.5–4.5) for residents, and 19 (IQR 14–26) for NPPs. We received a 76% response rate (52/68) (79% of attendings, 50% of residents, and 79% of NPPs) and 77% of respondents participated in at least two debriefings.

Emergency clinicians were primarily motivated to participate in these sessions to enhance their sense of community and connection (81%), support colleagues (75%), and better understand the emotional reactions of peers (71%). No other choices met our predefined threshold of >70% to be a significant factor and only 4% of emergency clinicians reported participating in order to process a specific clinical encounter. The clinicians reported four aspects specific to the debriefing process to be helpful: facilitators created a safe environment (98%); debriefing with members of the same role group (92%); facilitators were trusted colleagues (87%); and the Zoom platform was easy to use (81%). Among the surveyed programmatic aspects that respondents may have found unhelpful, none met our predefined threshold.

**Figure 1:**
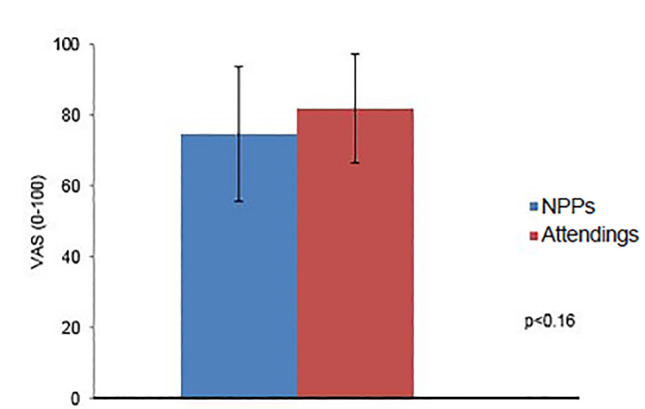
Comparative perceived value of debriefings between non-physician providers and attending physicians. *NPP*, non-physician provider; *VAS*, visual analogue scale.

The average perceived value of these sessions for emergency clinicians was 78.8 +/− 17.6 (IQR 73–89). There was no statistical difference in mean rating between attending physicians (81.9 +/− 15.7) and NPPs (74.8 +/− 19.5) (p = 0.16) (Figure 1).

**Figure 2:**
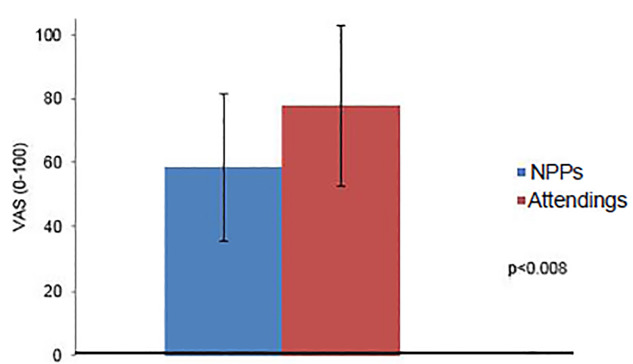
Comparative comfort speaking up during debriefings between non-physician providers and attending physicians. *NPP*, non-physician provider; *VAS*, visual analogue scale.

Emergency clinicians rated their comfort with speaking up during these debriefings to be 69.1 +/− 25.9 (IQR 52–93), and there was a statistical difference between attending physicians (77.8 +/− 25.0) and NPPs (58.5 +/− 23.6) (p = < 0.008) (Figure 2). Finally, emergency clinicians reported that debriefings contributed to a sense of connection with colleagues with an average 80.8 +/− 19.5 (IQR 69–96).

## DISCUSSION

We present a program to support the well-being of emergency clinicians during the coronavirus pandemic through video-based, emotion-oriented debriefings. Our results suggest that emergency clinicians are most interested in participating in this type of program to enhance their sense of community and connection with colleagues and understand emotional reactions of their peers, in comparison to less commonly identified reasons such as processing grief or a specific clinical encounter. Emergency clinicians sought opportunities to understand their peers’ emotional reactions to COVID-19 and support the range of emotional responses to the uncertainties and risks pervading both work and home environments. Debriefing also provided emergency clinicians with a platform to discuss unmet needs to improve self-care and build resilience.

Unlike critical-event debriefing and debriefing in simulation, there is less of a consensus around the best approach to debriefing clinicians on the emotional impact of a long-term public health crisis and occupational risk.^6^,^15^–^17^ We therefore employed an explorative approach to debriefing, focusing on empathy, compassion, and normalizing reactions. However, as the pandemic continues, debriefing specific emotions such as anxiety, guilt, isolation, and grief may become increasingly important at different phases of the crisis.^2^,^3^

Seventy-seven percent of respondents participated in two or more sessions. However, the voluntary nature of these debriefings may predispose our population to represent a subgroup of emergency clinicians who are more comfortable with sharing their emotional reactions with peers and showing vulnerability. This may influence our survey results and suggests that debriefing with peers may be a strategy to safeguard well-being for some but not all emergency clinicians. Recognizing this variability, we recommend that EDs interested in implementing a peer-based debriefing program incorporate it into a comprehensive approach to clinician wellness.

Finally, our finding that NPPs reported less comfort speaking up in a debriefing than attending physicians was unexpected. It is possible that low staff turnover of our attending group contributes to increased comfort with vulnerability. There may be less heterogeneity in professional experience for attendings than NPPs, influencing their perceived comfort with speaking up in these sessions. Hierarchy in clinical experience may also contribute to this finding. The attending leadership role may make speaking up easier, whereas NPPs are a clinically supervised group. Finally, the mean number of participants per session was higher for NPPs than attending physicians; this may also have contributed to the psychological safety of the debriefing environment. Further investigation is warranted as we grow the program to include other frontline emergency providers (eg, nurses and pharmacists). In the meantime, we plan to mitigate this potential factor by using Zoom’s breakout-room function.

## LIMITATIONS

Because it was a single-center study, the results of this intervention may have limited external validity. The process itself may have been influenced, either positively or negatively, by the facilitators’ relationship with the participants and previous interpersonal experiences, leading to a halo or millstone effect. Our survey did not account for external factors such as the level of ED preparedness and other wellness interventions by our administration that predate the pandemic. These may influence the way emergency clinicians experienced our debriefings. Further, our survey did not define “speaking up,” and this term may have been understood differently by participants, limiting interpretation of the results of this specific question.

Finally, our methodology did not allow us to investigate why few resident physicians volunteered to participate in our debriefings. Interventions implemented by the residency before the pandemic to support resident well-being, such as dedicated resident-only debriefing sessions during residency conference and a peer mentorship program, may have been effective and residents therefore did not elect to participate in our intervention.

## CONCLUSION

Emergency clinicians at our hospital reported that a video-based debriefing program was an acceptable and valuable intervention for supporting their emotional well-being during the initial phase of the coronavirus pandemic. The program provided participants with a platform to support each other and maintain a sense of community and connection despite social distancing. EDs should consider implementing a similar program to safeguard the emotional well-being of its clinician workforce as we move into subsequent phases of the pandemic.

## Supplementary Information
