# Supplementary material for: A Video-based Debriefing Program to Support Emergency Medicine Clinician Well-being During the COVID-19 Pandemic
Source: West J Emerg Med. 2020 Sep 25;21(6):88–92. doi: 10.5811/westjem.2020.8.48579 (PMC7673898; doi:10.5811/westjem.2020.8.48579)
Supplement: Supplementary file 1 [file wjem-21-88-s001.docx]

**Appendix A.** Facilitator guide: Emotion-oriented debriefing for ED Clinicians during COVID-19.

| **Stage** | **Duration (min)** | **Goals & Objectives** | **Suggested Language** |
| --- | --- | --- | --- |
| Opening | 3-5 | - Share overarching aim of the session with subjects: focus on emotional reactions to COVID-19; provide peer support; avoid problem-solving operational or clinical issues. - Describe confidentiality agreement. - Create psychologically safe debriefing environment. | - “Thank you for being here to support your peers. We’ll focus on sharing how we’re reacting to challenges at home and at work without trying to problem solve. - “If we identify an important issue, we - the facilitators - will bring it up with confidentially leadership on your behalf.” - “We ask that you keep the content of these sessions private. Please do not discuss any specific patient information. We will not record audio or video, but we will take written notes on major themes.” - “We know it can be hard to discuss emotional reactions. If at any time you need to take a break, you can return to the Zoom lobby, no questions asked. You can also send either facilitator a private direct message through the chat function.” - “Before we get started, which questions can we help answer?” |
| Discussion | 45 | - Normalize reactions. - Facilitate discussion about challenges in clinical and home environments; steer toward empathy and emotional validation. - Guide subjects through reflection on shared experiences, silver-linings, and gratitude statements. | - “Let’s talk about something that has challenged you (at home/work) this week.” - “You mentioned ___, can you say a little bit more about how you felt at that time.” - “Thank you for sharing that; you’re not the only one who has shared a similar reaction with us.” - “I think many of us would feel similarly if we were in your shoes.” - “It’s normal to experience these moments differently. Has anyone else had a different reaction when thinking about ____?” |
| Closing | 5-10 | - Show subjects respect by acknowledging the hour is nearing its end. - Open up the discussion to any subject who has not had an opportunity to discuss something important to them. - Give subjects a sense of closure by helping them identify 1-2 take-aways. | - “We’re nearing the end of our debriefing. Anyone who needs to leave a few minutes early for any reason, please feel free to do so at any point.” - “We want to make sure everyone has an opportunity to share what’s on their mind. Any last reactions?” - “We’ve talked about __, __, and __. What other points from the session resonate with the group?” - “Thank you for being here. We know it isn’t easy to share emotional reactions. We’ve placed a link to Well-Being Resources provided by Partners in the group chat; we recommend taking a look after the session.” |
