# Supplementary material for: A Video-based Debriefing Program to Support Emergency Medicine Clinician Well-being During the COVID-19 Pandemic
Source: West J Emerg Med. 2020 Sep 25;21(6):88–92. doi: 10.5811/westjem.2020.8.48579 (PMC7673898; doi:10.5811/westjem.2020.8.48579)
Supplement: Supplementary file 2 [file wjem-21-88-s002.docx]

**Appendix B**

**Survey Design**

To create this survey, study team members (DM, JT) reviewed previous surveys used to evaluate debriefings related to well-being and wellness in healthcare. Based on these limited results, study team members (DM, JT) created questions that focused on debriefing participants’ experience with the program. We solicited feedback from remaining study authors and incorporated recommendations into the final survey.

**Survey on Emotion-Oriented Debriefing Experience.**

* Required

**1.** What is your medical profession? * (select one) a) Physician b) Resident Physician c) Advanced Practice Provider (PA or NP)

**2.** How many COVID-19 emotion-oriented debriefings for MGH ED clinicians have you participated in? * (select one) a) 1 ; b) 2-3 ; c) 4 or more

**3.** What was your original motivation for participating in these debriefings? Please select all that apply.

1. To feel more supported/cared for
2. To better understand my own emotional reactions to coronavirus pandemic
3. To better understand the emotional reactions of my peers to coronavirus pandemic
4. To enhance my sense of community and connection
5. To process grief
6. To process a specific clinical encounter
7. To support my colleagues
8. Other (please describe):

**4.** How helpful did you find these debriefings?

Please mark a point on the line between 0 and 100, with 0 being “not at all helpful” and 100 being “extremely helpful.”

0 100

**5.** If you found these debriefings to be helpful, which of the following made it so? Please select all that apply:

1. The objectives of the debriefing were clear.
2. The facilitators created a safe environment for sharing emotions.
3. The debriefing participants were members of my role group.
4. I found the Zoom platform easy to use.
5. The sessions provided enough time to process my emotions.
6. Having these meetings scheduled in advance gave me a sense of comfort.

**6.** How comfortable did you feel speaking up during these debriefings?

Please mark a point on the line between 0 and 100, with 0 being “not at all comfortable” and 100 being “extremely comfortable.”

0 100

**7.** How helpful were these debriefings in terms of contributing to your sense of connection with your colleagues?

Please mark a point on the line between 0 and 100, with 0 being “not at all helpful” and 100 being “extremely helpful.”

0 100

**8.** If you found these debriefings to be unhelpful, which of the following made it so? Please select all that apply:

1. Intimidated by debriefing in front of peers
2. Poorly organized
3. Made me feel worse about the pandemic
4. Did not help me process my own emotional reactions
5. Did not provide me with a sense of connection with peers
6. Did not give you avenues to discuss this topic with professional resources
7. Difficulty using the Zoom platform
8. Not applicable; I found debriefings to be helpful.

**9.** If you would like to add anything further about the debriefings and how it has affected you, please share:

**10.** If you would like to provide feedback on how we might improve these debriefings, please share:
